# Automated development of the contrast–detail curve based on statistical low‐contrast detectability in CT images

**DOI:** 10.1002/acm2.13719

**Published:** 2022-07-09

**Authors:** Choirul Anam, Ariij Naufal, Toshioh Fujibuchi, Kosuke Matsubara, Geoff Dougherty

**Affiliations:** ^1^ Department of Physics Faculty of Sciences and Mathematics Diponegoro University Semarang Central Java Indonesia; ^2^ Department of Health Sciences Faculty of Medical Sciences Kyushu University Fukuoka Japan; ^3^ Department of Quantum Medical Technology Faculty of Health Sciences Institute of Medical Pharmaceutical and Health Sciences Kanazawa University Kanazawa Japan; ^4^ Department of Applied Physics and Medical Imaging California State University Channel Islands Camarillo California USA

**Keywords:** image quality, low‐contrast detectability, minimum detectable contrast

## Abstract

**Purpose:**

We have developed a software to automatically find the contrast–detail (*C*–*D*) curve based on the statistical low‐contrast detectability (LCD) in images of computed tomography (CT) phantoms at multiple cell sizes and to generate minimum detectable contrast (MDC) characteristics.

**Methods:**

A simple graphical user interface was developed to set the initial parameters needed to create multiple grid region of interest of various cell sizes with a 2‐pixel increment. For each cell in the grid, the average CT number was calculated to obtain the standard deviation (SD). Detectability was then calculated by multiplying the SD of the mean CT numbers by 3.29. This process was automatically repeated as many times as the cell size was set at initialization. Based on the obtained LCD, the *C*–*D* curve was obtained and the target size at an MDC of 0.6% (i.e., 6‐HU difference) was determined. We subsequently investigated the consistency of the target sizes for a 0.6% MDC at four locations within the homogeneous image. We applied the software to images with six noise levels, images of two modules of the American College of Radiology CT phantom, images of four different phantoms, and images of four different CT scanners. We compared the target sizes at a 0.6% MDC based on the statistical LCD and the results from a human observer.

**Results:**

The developed system was able to measure *C*–*D* curves from different phantoms and scanners. We found that the *C*–*D* curves follow a power‐law fit. We found that higher noise levels resulted in a higher MDC for a target of the same size. The low‐contrast module image had a slightly higher MDC than the distance module image. The minimum size of an object detected by visual observation was slightly larger than the size using statistical LCD.

**Conclusions:**

The statistical LCD measurement method can generate a *C*–*D* curve automatically, quickly, and objectively.

## 1 INTRODUCTION

Computed tomography (CT) is used in health facilities for diagnosis, screening, image‐guided radiotherapy, and image‐guided surgery.^1^, ^2^ Because CT utilizes ionization radiation, the use of CT should be optimized, that is, the radiation dose kept to a minimum, while maintaining image quality.^3^, ^4^, ^5^, ^6^ Therefore, quantifying the image quality is important. One of the key performance indicators of CT image quality is its ability to differentiate low‐contrast lesions.^7^, ^8^ This is reported to be one of the main advantages of CT compared with other medical imaging modalities.^9^ The most common metric to differentiate low‐contrast lesions is the contrast‐to‐noise ratio.^10^, ^11^ However, this metric is not sufficient for identifying the details of imaging low‐contrast lesions because it does not take into account image frequency, which can affect detectability.^12^ A more robust assessment is quantified in metrics of low‐contrast detectability (LCD).

Many studies have explored LCD, including those related to CT dose optimization.^13^, ^14^, ^15^, ^16^, ^17^ The phantom module used in identifying LCD can generally be divided into two categories. The first category uses materials with slightly different densities, as in the Catphan phantom (i.e., CTP515 module). Because CT detects contrast differences lower than 1%,^13^ such phantoms are designed with high density accuracy making them very expensive. The second category utilizes the partial‐volume effect. Such phantoms use a material whose density is close to the density of the background. The partial‐volume effect between the object and the background results in an object with almost similar CT number as the background. However, the partial‐volume effect strongly depends on the slice thickness, so that the LCD measurement is dependent on the accuracy of the nominal thickness of the reconstructed slice image. In addition, LCD measurements on the low‐contrast object are generally identified based on the average scores of selected human observers.^18^, ^19^ The LCD measurement is determined based on the smallest object that can still be seen at a certain level of contrast. Visual investigation by human observers of LCD is complex, time‐consuming, and subjective. As a result, they are subject to large inter‐ and intra‐observer variability.^20^ Moreover, human observer studies can be biased due to the observer knowing the pattern of the objects in the phantom.^21^

In order to reduce these complexities, a statistical LCD measurement method was introduced.^22^ Statistical LCD can be measured on homogeneous phantom images by computer. The region of interest (ROI) consists of a grid comprising cells of the same size. The mean CT number for each cell in the grid is calculated. The standard deviation (SD) of all the means is calculated and multiplied by 3.29 so that the observed low‐contrast objects can be identified from the background with a 95% confidence level (CL).^13^

The statistical LCD measurement feature sometimes exists within CT scanners in the form of built‐in tools, but it is limited to particular vendors through the operator panel.^23^ In addition, the built‐in software from certain vendors can only be used for particular phantoms. Vendor‐independent software to analyze the LCD has been developed by Chacko et al.^24^ However, it is only able to measure LCD with one particular cell size. The measurement process must be repeated manually with different cell (object) sizes to be able to produce a contrast–detail (*C*–*D*) curve. After the LCD values for each cell size are obtained, a *C*–*D* curve can be plotted,^25^ and a trend line drawn to connect the various cell sizes. The process is less effective and tedious.

To address these problems, we developed a software to automatically find the *C*–*D* curve based on statistical LCD and measure the target size at the minimum detectable contrast (MDC) of 0.6% (i.e., 6‐HU difference) from images of various phantoms (that have uniform regions) and images from various scanners. The algorithm is implemented with simple initial settings and needs only a single click to accomplish all tasks. The user sets the ROI in a flexible size range as well as the number of ROIs that are arranged in a grid. The software generates a *C*–*D* curve automatically with an embedded trend line that follows the power‐law fit.^25^

It is worth noting that statistical LCD assumes that both object and background noises follow a Gaussian distribution. This assumption may not apply to CT images reconstructed by iterative reconstruction (IR), that is, an alternative reconstruction technique commonly used today in clinical setting. A previous study^25^ evaluated the blending fraction of one of the IR algorithms, that is, adaptive statistical iterative reconstruction (ASIR) on the specified LCD. It was reported that the noise distribution is normal (i.e., Gaussian) in the presence of ASIR.^25^ In the current work, the developed software was used to investigate the *C*–*D* curves for images reconstructed using FBP at four locations within the homogeneous image, images with six noise levels, images of two modules of the American College of Radiology (ACR) CT phantom,^26^ images of four different phantoms, and images of four different CT scanners. The target sizes at a 0.6% MDC based on the statistical LCD were compared with human observers.

## 2 METHODS

### 2.1 Statistically defined LCD

**FIGURE 1:**
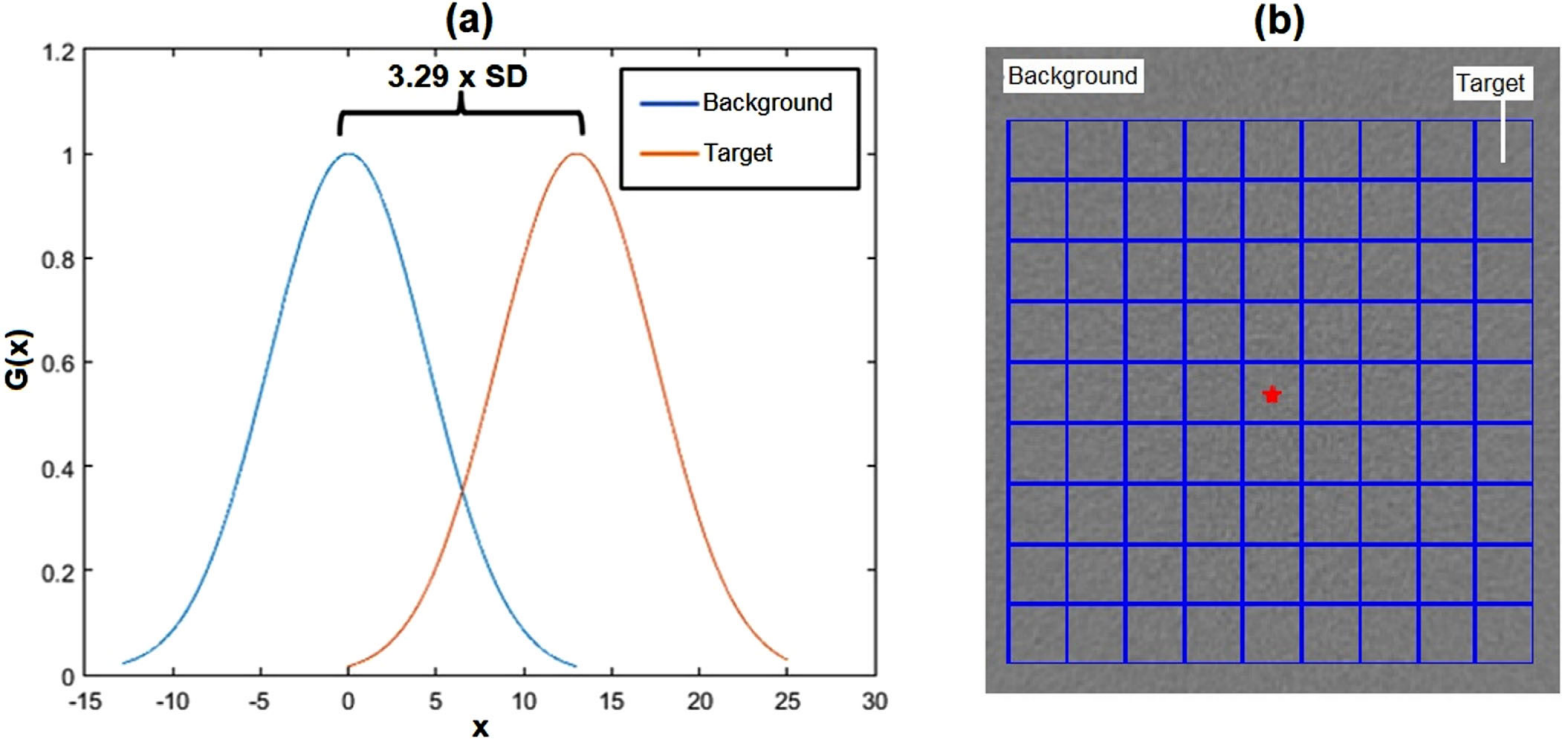
(a) Statistical low‐contrast detectability (LCD) is determined by 3.29 × standard deviation (SD) of distributions of average computed tomography (CT) numbers of the object (target) and the background. (b) Grid region of interest (ROI) for statistically‐defined LCD (SD‐LCD) measurement

The statistically defined LCD (SD‐LCD) determination is based on the assumption that an object with a certain size and density can be distinguished from its background if the object contrast is higher than the noise level on the same spatial scale.^27^, ^28^ The statistical method assumes that the mean CT number of many low‐contrast objects of the same size follows a Gaussian distribution (Figure 1a). (The mean CT number of the background also follows the same distribution.) At the same input scan parameters, the average CT number of both the object and the background will have the same SD. If the midpoint is shifted to separate the two curves between the object and the background leaving a 5% false‐positive rate (95% CL), then the shift will be fulfilled at 3.29 × SD.^25^ Statistically, object size considerations can be approached using a grid‐shaped ROI where the cell size represents the object size (Figure 1b). If the grid ROI is placed in a uniform region of the image, the distributions of the pixel mean of each cell will have the same shape but need to be increased from the average of the background pixels by a certain amount, so that objects of that size can be distinguished from the background. SD‐LCDs are usually expressed in resolvable contrasts (MDCs) for various lesion sizes.^13^

### 2.2 *C*–*D* curve based on statistical LCD

#### 2.2.1 Initialization

**FIGURE 2:**
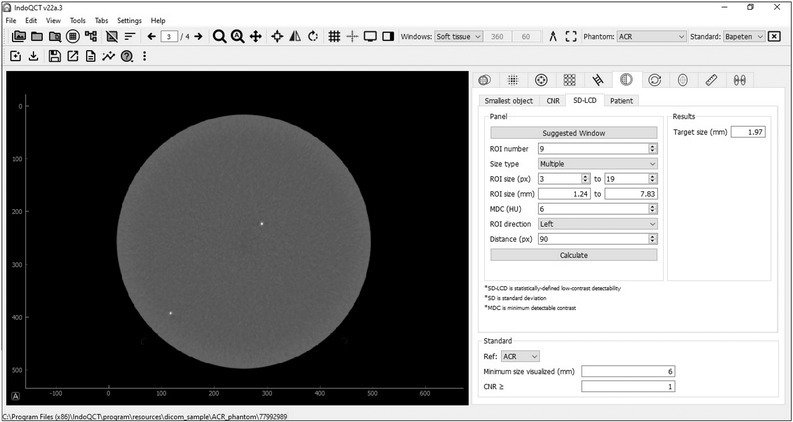
Graphical user interface (GUI) of statistically defined low‐contrast detectability (SD‐LCD) measurement

The software to automatically find the *C*–*D* curve based on statistical LCD was written using the Python programming language. Figure 2 depicts a graphical user interface (GUI) that is useful for configuring initial parameters. At the initialization stage, the GUI panel collects information about a number and range size of cells set by the user. For multiple cell sizes, the required size ranges from the smallest to the largest in steps of 2 pixels. The center coordinates of the cell grid, which is at the center of the phantom image, were calculated using the centroid of the segmented binary image (Equation 1).^27^, ^28^

(1) $$\begin{array}{c} X_{c}=\frac{1}{\left|R\right|}\;.\sum_{\left(i,j\right)\in R}i\\ Y_{c}=\frac{1}{\left|R\right|}\;.\sum_{\left(i,j\right)\in R}j \end{array}$$

where *X_c_* and *Y_c_* are the center coordinates. *R* is a region of a binary image that can be interpreted as a two‐dimensional distribution of foreground.

#### 2.2.2 Creating ROI grids for multiple cell sizes

In the implementation of multiple cell sizes, the algorithm iterated according to the number of cell sizes ranging from the smallest to the largest cell sizes in steps of 2 pixels. In the first iteration, the algorithm defined one cell on the grid equal to the first cell size plus half a cell (which is half of the first cell size). The coordinates of the beginning (top left) and the end (bottom right) were determined by

(2) $$\begin{array}{c} Y_{i}=Y_{c}-\left(d\times 0.5n_{\mathrm{roi}}\right)-0.5d\\ X_{i}=X_{c}-\left(d\times 0.5n_{\mathrm{roi}}\right)-0.5d\\ Y_{f}=Y_{c}+\left(d\times 0.5n_{\mathrm{roi}}\right)+0.5d\\ X_{f}=X_{c}+\left(d\times 0.5n_{\mathrm{roi}}\right)+0.5d \end{array}$$

where *X_i_* and *Y_i_* are the initial coordinates of the grid, *X_f_* and *Y_f_* are the final coordinates of the grid, and *X_c_* and *Y_c_* are the coordinates of the center of the phantom. *d* is the cell size in pixels and *n*_roi_ is the cell number.

The grid was created by iterating from the initial coordinates to the final coordinates of the grid in increments of the first cell size. The pixel mean in HU was obtained by collecting the pixel values from the HU image treated as an array of data as shown in the following equation:

(3) $$\bar{\mathrm{HU}}=\;\frac{1}{d^{2}}\sum_{i,j}^{d}X_{i,j}$$

where *i* and *j* are the initial coordinates of each cell in the grid, and *d* is the size of the ROI in pixels.

For each cell in the grid, a similar process was carried out. We then measured the mean CT number of all the cells. From these means, the SD was calculated and multiplied by 3.29 to obtain the detectability for a single cell size.^25^ This series of processes were then repeated for the next cell size and so on, until the largest size set by the user at the initialization stage. This gave the detectability for each cell size.

**FIGURE 3:**
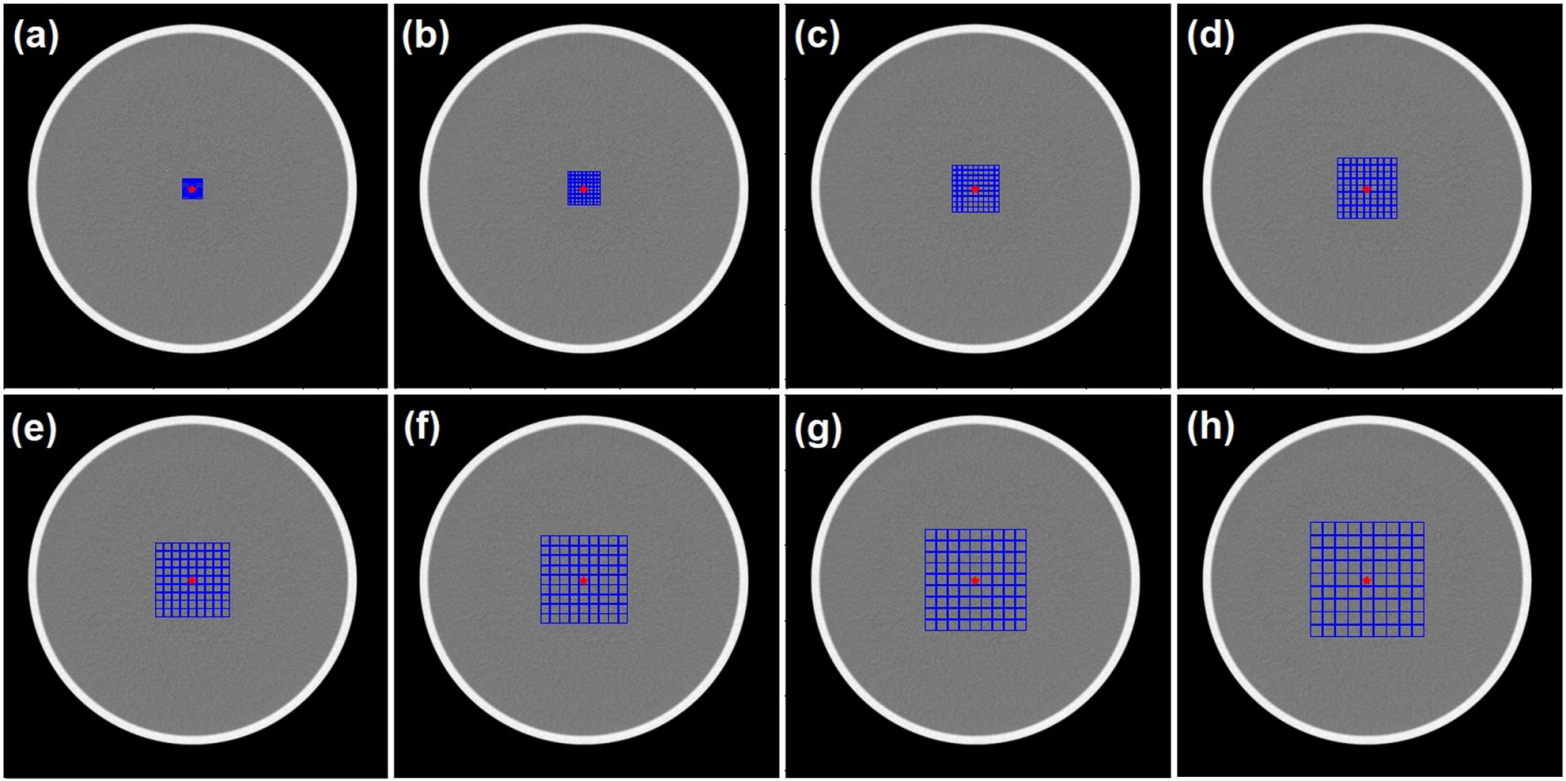
Multiple grid region of interests (ROIs) for statistical low‐contrast detectability (LCD) measurement with cell sizes of (a) 3 × 3 pixels, (b) 5 × 5 pixels, (c) 7 × 7 pixels, (d) 9 × 9 pixels, (e) 11 × 11 pixels, (f) 13 × 13 pixels, (g) 15 × 15 pixels, and (h) to 17 × 17 pixels. The cell number is 9 × 9. The red marker indicates the centroid coordinates of the phantom image.

**FIGURE 4:**
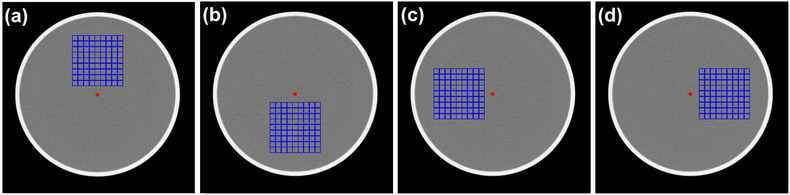
Multiple grid region of interests (ROIs) for statistical on low‐contrast detectability (LCD) measurement with cell sizes of 15 × 15 pixels and cell number 9 × 9 for different locations (the center of ROI was moved 90 pixels from the centroid): (a) up, (b) down, (c) left, and (d) right positions

The range cell size can be set by the user (viz., from 3 to 23 pixels) depending on the size of the phantom and field of view of the image. The cell number can also be set by user (viz., 9 × 9) (Figure 3). The star‐shaped red marker marks the coordinates of the phantom center in the image as an initial reference. Each cell is marked at the edges with a blue marker so that it forms grid lines. There was an additional option to shift the center of the grid from the phantom centroid to avoid inhomogeneity within the image or to find the *C*–*D* curve from a different location within the image (Figure 4).

The cell size in pixels was converted to mm to make it easier to identify the size of the lesion according to pixel size. The cell size in mm was adjusted from pixel sizes by the pixel spacing value obtained from the DICOM info.

#### 2.2.3 *C*–*D* curve

**FIGURE 5:**
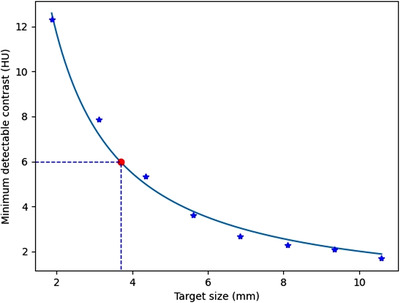
An example of the contrast–detail (*C*–*D*) curve with power‐law fit and a dashed line indicating 0.6% minimum detectable contrast (MDC) and its target size

Each detectability data and cell size was stored in two different variables, and the cell size and detectability were used to construct the *C*–*D* curve. The trend line was added using interpolation according to the power‐law fit as shown in the following equation^27^:

(4) $$Y=kX^{\alpha }$$

where *X* and *Y* are the variables of interest, *α* is the law's exponent, and *k* is a constant. Once the LCD measurement is activated, the process is begun and the *C*–*D* curve is automatically generated. The MDC and its target size can be automatically measured from the *C*–*D* curve. In the current study, there was a highlight point at a 0.6% MDC. However, the MDC can be set to a value other than 0.6% by the user.^29^ Figure 5 shows an example of a *C*–*D* curve with a dashed line indicating a 0.6% MDC and its target size.

### 2.3 Images of phantoms

**FIGURE 6:**
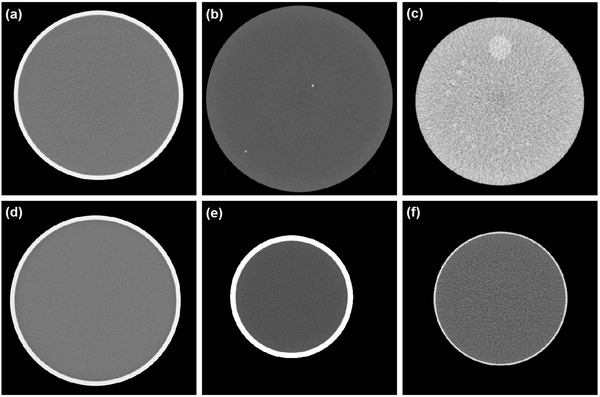
Axial images of (a) Catphan (CTP486) phantom, (b) distance module of American College of Radiology (ACR) computed tomography (CT) phantom, (c) low‐contrast module of ACR CT phantom, (d) GE CT QA phantom, (e) Philips CT phantom, and (f) Hitachi CT phantom

We subsequently investigated the consistency of the target sizes with a 0.6% MDC at four locations (i.e., up, down, left, and right positions) within the homogeneous image of the Catphan phantom (The Phantom Laboratory, NY, USA) (Figure 6a). We wanted to determine whether the target size at a 0.6% MDC was different in the four locations. This is because the target size value may be affected by noise level and the noise level depends on the measurement position. The phantom consisted of water with a diameter of 21 cm. The phantom was scanned using the GE LightSpeed Pro 32. The scan parameters are tabulated in Table 1.

**TABLE 1:** Scan parameters for various slice thicknesses and positions

Parameters	Variation of ROI position	Variation of noise level
Scanner	GE LightSpeed Pro 32	Philips MX 16‐slice
Phantom	Catphan	ACR
Voltage (kVp)	120	120
Tube current (mA s)	700	300
Pitch	0.96875	0.6713
Slice thickness (mm)	1.25	1.5, 3, 5, 6, 7, 10
Convolution kernel	Standard	SB
Rotation time (s)	1	1
Scan mode	Helical	Helical

Abbreviations: ACR, American College of Radiology; ROI, region of interest.

The software was used to measure the MDCs based on statistical LCD from different homogeneous images from six noise levels within the same module, two modules within the same phantom, four different phantoms, and four different CT scanners to demonstrate the universality of our software.

The impacts of six different noise levels on the *C*–*D* curve and the 0.6% MDC were measured from images of the distance module of the ACR phantom (Gammex Inc., USA) (Figure 6b). The variation of noise level was achieved using slice thicknesses of 1.5, 3, 5, 6, 7, and 10 mm. The distance module consists of a uniform tissue‐equivalent material (∼0 HU) with two very small cubes of radiopaque steel balls each of diameter ∼0.28 mm. The phantom was scanned using a Philips MX 16‐slice. The scan parameters are tabulated in Table 1.

The *C*–*D* curve and the 0.6% MDC from the distance module were compared to those from the low‐contrast module of the ACR phantom (Figure 6c). The low‐contrast module of the ACR phantom consists of a series of cylinders of different diameters, namely, 2, 3, 4, 5, and 6 mm. The space between cylinders is equal to the cylinder diameter. All cylinders have a contrast of 6‐HU difference from the background. The background material has a mean CT number of ∼90 HU.

Subsequently, the software was used to evaluate the *C*–*D* curves and 0.6% MDCs from images of four different phantoms: Catphan, GE CT QA (Figure 6d), Philips (Figure 6e), and Hitachi (Figure 6f) phantoms. The four phantoms were homogeneous phantom made from water. The phantoms had different diameters. The diameters were 214, 215, 197, and 305 cm for Catphan, GE CT QA, Philips, and Hitachi phantoms, respectively. The phantoms were scanned with different input parameters (Table 2).

**TABLE 2:** Scan parameters for various phantoms

Parameters	Phantom			
	Catphan	GE CT QA	Philips	Hitachi
Scanner	GE LightSpeed Pro 32	GE Revolution EVO	Philips Brilliance 16	Hitachi ECLOS
Voltage (kVp)	120	120	120	120
Tube current (mA s)	700	155	225	250
Pitch	0.96875	0.53125	0.563	–
Slice thickness (mm)	1.25	5	2	1.25
Convolution kernel	Standard	Standard	UB	32
Rotation time (s)	1	0.8	0.75	1
Scan mode	Helical	Helical	Helical	Axial

Abbreviations: CT, computed tomography.

Finally, the *C*–*D* curves and 0.6% MDCs were measured from images of the ACR phantom scanned with four different scanners of Hitachi SUPRIA, Siemens Emotion 16, Siemens SOMATOM go.Now, and Toshiba Alexion. The input parameters were tabulated in Table 3.

**TABLE 3:** Scan parameters for various scanners

	Scanner			
Parameters	Hitachi SUPRIA	Siemens Emotion 16	Siemens SOMATOM go.Now	Toshiba Alexion
Voltage (kVp)	120	110	130	120
Tube current (mAs)	200	300	118	150
Pitch	–	–	–	0.688
Slice thickness (mm)	10	8	0.8	1
Convolution kernel	11	H31s	Hr36f, 3	FC68
Rotation time (s)	1	1	1.666	1.5
Scan mode	Axial	Axial	Axial	Helical

### 2.4 Visual evaluation

The results of the automated measurement of the *C*–*D* curves and 0.6% MDCs based on the statistical LCD were compared to those from a human observer. For visual evaluation, we used an image of a low‐contrast module of the ACR CT accreditation phantom. We observed visually the low‐contrast module at a window width of 100 HU and a window level of 100 HU for all noise level variations. In this task, we investigated the smallest object that can still be seen.^29^ The object size was matched with the closest interpolation results at 6‐HU contrast, according to a previous study.^29^

## 3 RESULTS

### 3.1 Impact of different positions on *C*–*D* curves

**FIGURE 7:**
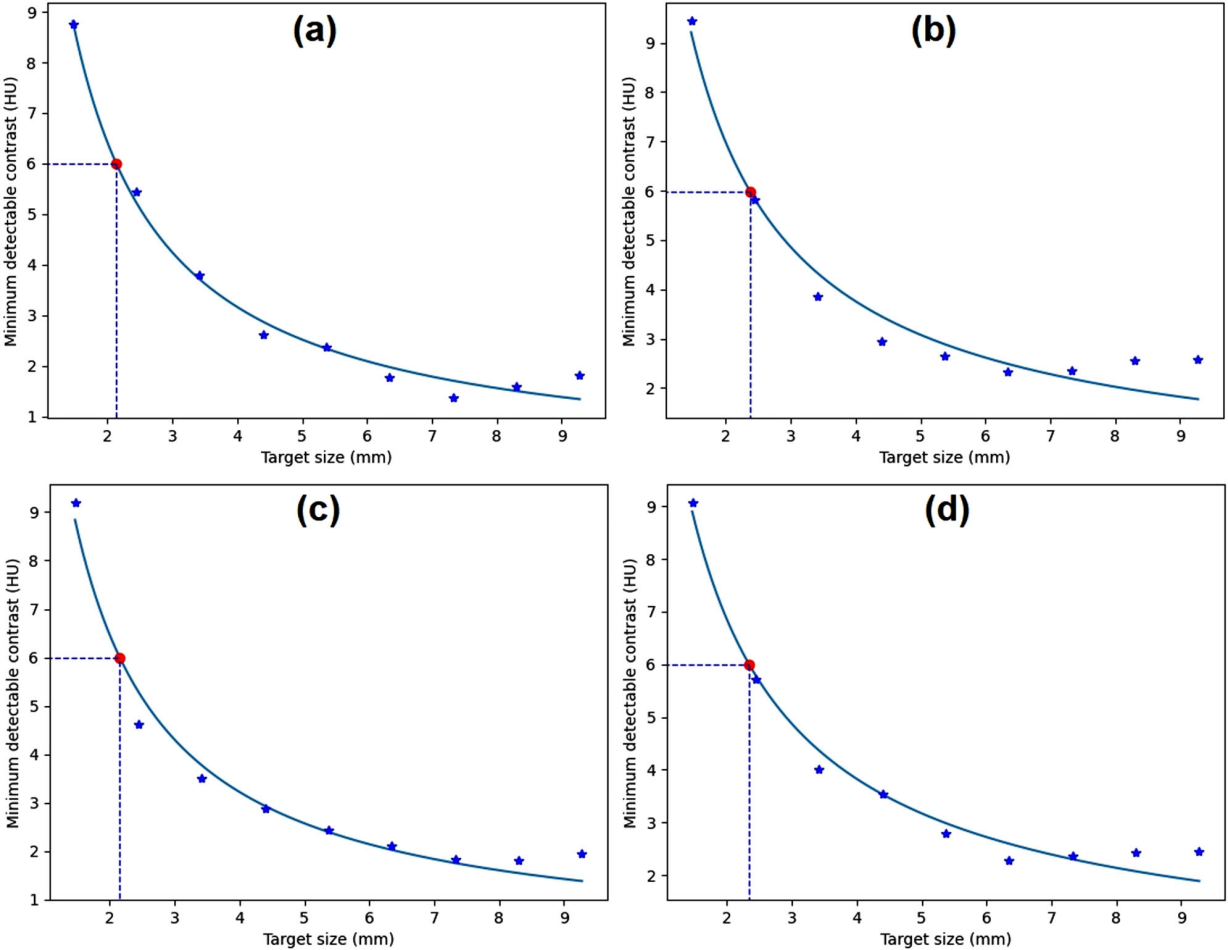
Contrast–detail (*C*–*D*) curves generated from homogeneous module (CTP486) of Catphan phantom for various region‐of‐interest (ROI) positions of distance 90 pixels: (a) up, (b) down, (c) left, and (d) right positions

Figure 7 shows the *C*–*D* curves generated from a homogeneous image of the Catphan phantom in four ROI positions, that is, up, down, left, and right positions. It shows that there is a decrease in MDC as the size of the lesion increases. Smaller objects require a larger MDC to be distinguished from the background. The *C*–*D* curves follow the power‐law fit. Variations of ROI position give comparable *C*–*D* curves and target size at the 0.6% MDC. The point highlight represents the target size at the 0.6% MDC, and is related to the contrast of the material in the module.^28^ This highlight is obtained from the point closest to the power‐law interpolation in the data. The target sizes at the 0.6% MDC were 2.31, 2.14, 2.37, 2.15, and 2.34 mm at center, up, down, left, and right positions, respectively. This shows that the maximum difference of the target size was very small (0.17 mm).

### 3.2 Impact of noise on *C*–*D* curves

**FIGURE 8:**
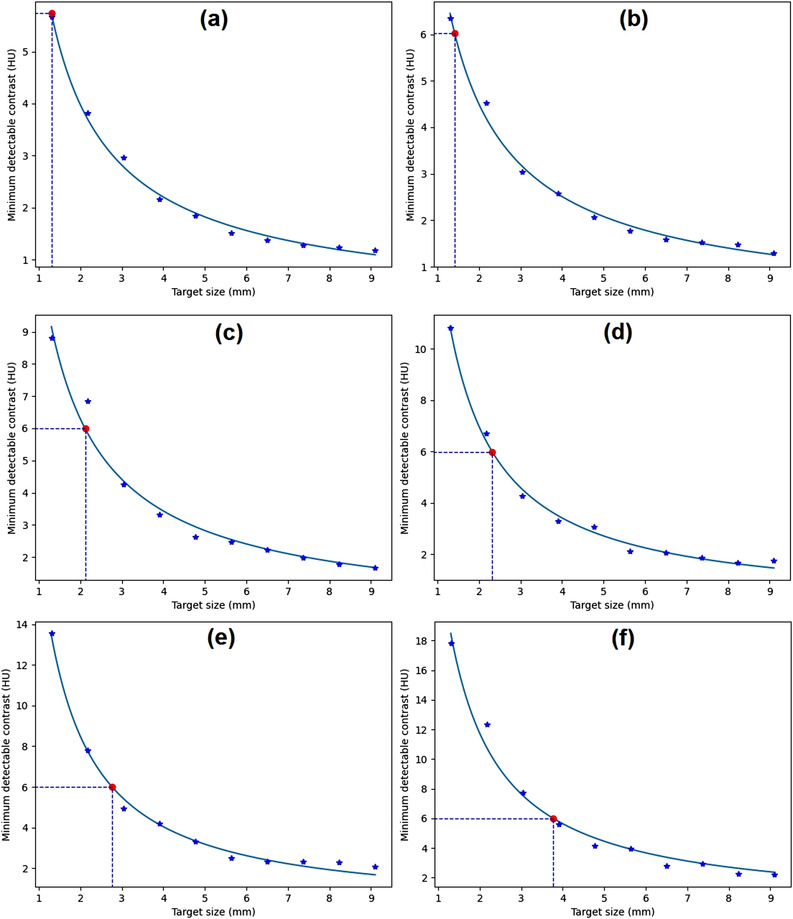
The contrast–detail (*C*–*D*) curves generated from images of different noise levels: (a) 2.4 HU, (b) 3.0 HU, (c) 3.8 HU, (d) 4.1 HU, (e) 4.8 HU, and (f) 7.6 HU

Noise level variation was achieved by the variation of slice thickness. In the distance module of the ACR CT phantom, the noise levels were 2.4 ± 0.3, 3.0 ± 0.4, 3.8 ± 0.3, 4.1 ± 0.4, 4.8 ± 0.6, and 7.6 ± 1.1 HU for slice thicknesses of 10, 7, 6, 5, 3, and 1.5 mm, respectively. The *C*–*D* curves for noise level variation are depicted in Figure 8. It is clear that the MDC increases with increasing noise level. The target sizes (for 0.6% MDC) were 1.3, 1.4, 2.1, 2.3, 2.8, and 3.8 mm for noise levels from 2.4 ± 0.3 to 7.6 ± 1.1 HU, respectively.

### 3.3 Impact of different modules on *C*–*D* curves

**FIGURE 9:**
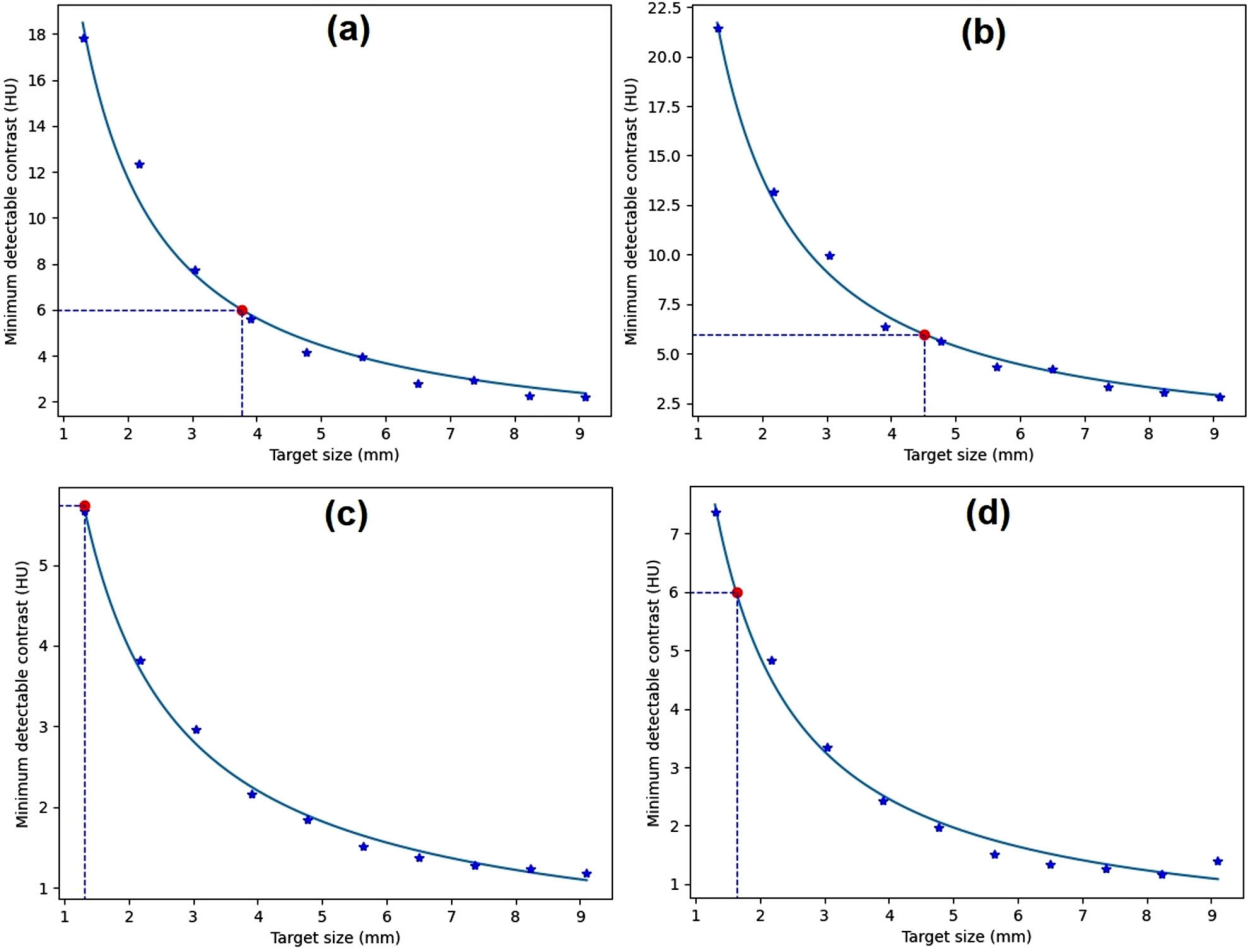
The contrast–detail (*C*–*D*) curves generated from images of different modules of the American College of Radiology (ACR) computed tomography (CT) phantom: (a) distance module (slice thickness of 1.5 mm), (b) low‐contrast module (slice thickness of 1.5 mm), (c) distance module (slice thickness of 10 mm), and (d) low‐contrast module (slice thickness of 10 mm)

Figure 9 shows comparisons of *C*–*D* curves from the distance and low‐contrast modules of the ACR CT phantom (for slice thicknesses of 1.5 and 10 mm). It shows that the low‐contrast module image had a slightly higher MDC than the distance module image for the same slice thickness and target size. The target sizes (for 0.6% MDC) for the low‐contrast module were 4.5 and 1.6 mm for slice thicknesses of 1.5 and 10 mm, whereas the target sizes for distance module were 3.8 and 1.3 mm for slice thicknesses of 1.5 and 10 mm. The noise levels for the distance module were 7.6 ± 1.1 and 2.4 ± 0.3 HU for slice thicknesses of 1.5 and 10 mm, whereas the noise levels for the low‐contrast module were 10.3 ± 0.6 and 3.3 ± 0.1 HU for slice thicknesses of 1.5 and 10 mm.

### 3.4 Impact of different phantoms on *C*–*D* curves

**FIGURE 10:**
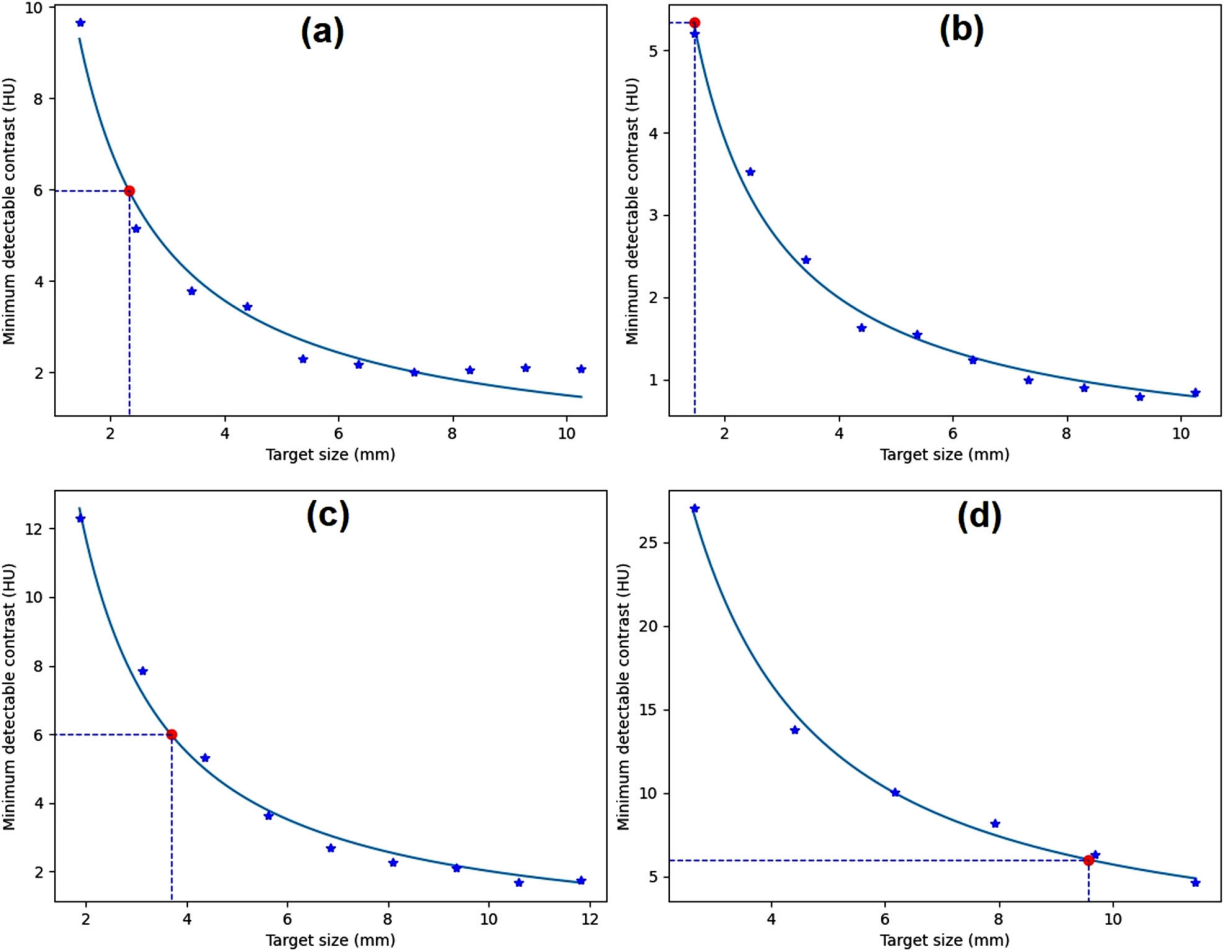
The contrast–detail (*C*–*D*) curves generated from the homogeneous module of (a) Catphan (CTP486) phantom, (b) GE computed tomography (CT) QA phantom, (c) Philips CT phantom, and (d) water phantom

Figure 10 shows the *C*–*D* curves generated from homogeneous images from various phantoms. The target sizes at the 0.6% MDC were 2.3, 1.5, 3.7, and 9.6 mm, and the noise levels were 4.7, 2.6, 6.3, and 21.1 HU for the Catphan, GE, Philips, and Hitachi phantoms, respectively.

### 3.5 Impact of different scanners on *C*–*D* curves

**FIGURE 11:**
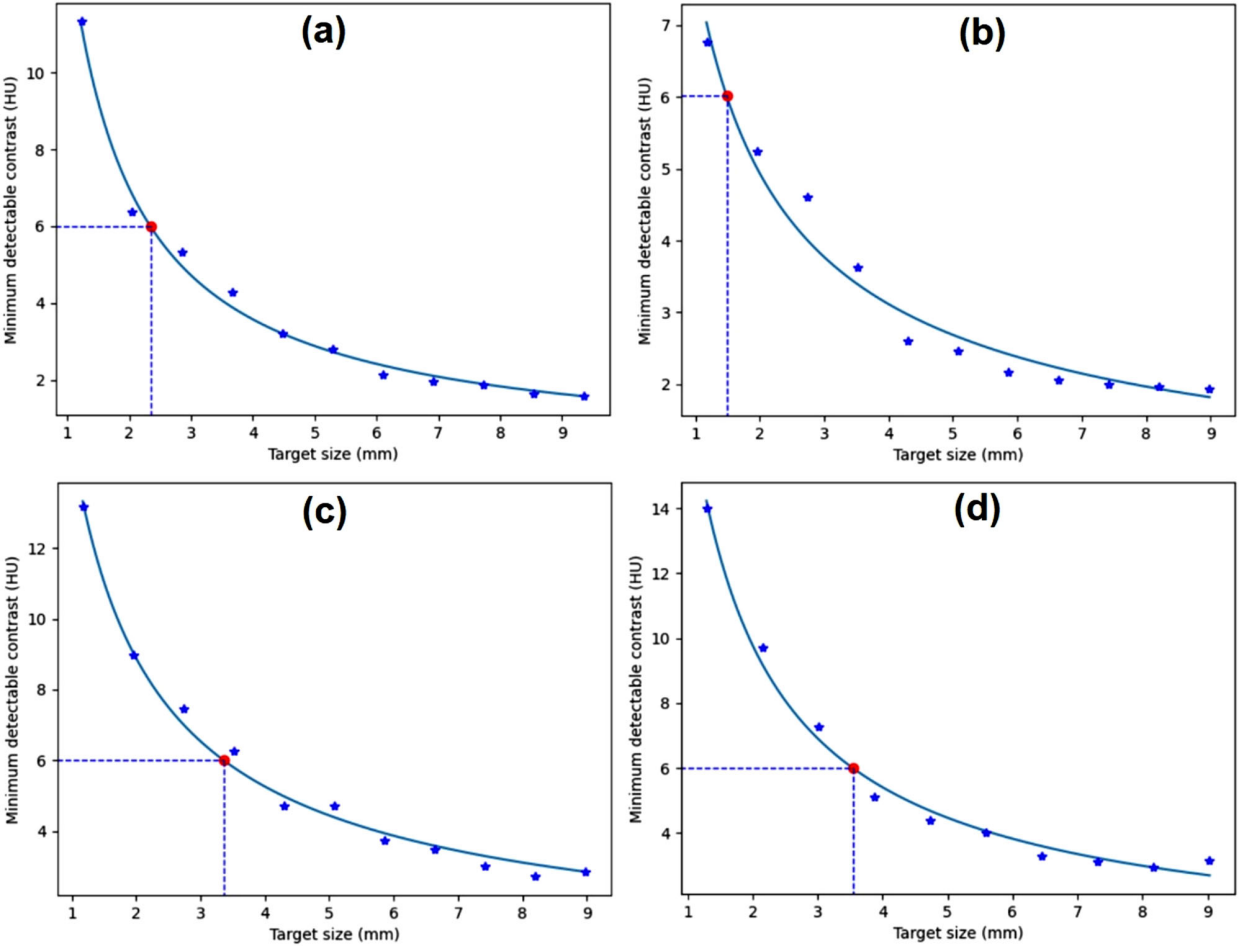
The contrast–detail (*C*–*D*) curves generated from distance module of American College of Radiology (ACR) computed tomography (CT) phantom scanned with (a) Hitachi SUPRIA, (b) Siemens Emotion 16, (c) Siemens SOMATOM go.Now, and (d) Toshiba Alexion

Figure 11 shows the *C*–*D* curves generated from the distance module of the ACR CT phantom scanned with various scanners. The target sizes at 0.6% MDC were 2.4, 1.6, 3.4, and 3.6 mm, and the noise levels were 3.9, 3.1, 5.6, and 5.8 HU for (a) Hitachi SUPRIA, (b) Siemens Emotion 16, (c) Siemens SOMATOM go.Now, and (d) Toshiba Alexion, respectively.

### 3.6 Comparison to visual evaluation

Visual observations by the authors were conducted to compare with the results of the automated *C*–*D* curves. The image used was a low‐contrast module image containing cylindrical objects of various sizes and with windowing settings as recommended in the manual (window width = 100 HU and window level = 100 HU).^25^ The results of 0.6% MDC by visual observation are tabulated in Table 4. The size of the object detectable by visual observation was slightly larger compared to the results of the automated method based on statistical LCD at the same contrast of 6 HU.

**TABLE 4:** Results of visual observations on the 0.6% minimum detectable contrast (MDC) identified using the low‐contrast module

Noise level (HU)	Target size (mm)	
	Visual observation	Statistical LCD
10.3	5.0	4.5
6.5	4.5	2.8
5.5	4.0	2.4
5.0	3.5	2.3
3.8	3.0	1.8
3.3	2.0	1.6

Abbreviation: LCD, low‐contrast detectability.

## 4 DISCUSSION

Quality control of LCD of small objects in CT images is important. This is a relatively difficult task and can be problematic, especially when it comes to detecting lesions that are nearly the same density as the surrounding tissue. This is because it depends on the ability of an observer to distinguish an object that has a CT number similar to the background. We developed a statistical calculation that is validated by observations on low‐contrast phantoms.

LCD measurement can be limited due to the absence of phantoms that have a low‐contrast module. Even with such a phantom, the identification of low‐contrast objects is carried out manually by human observers with their own subjectivity.^7^, ^32^ One factor that can increase the subjectivity of human observer measurement is observer bias, where the observer already knows the shape and pattern of the material embedded in the module.^33^ Other biases come from the experience of the observer, as well as the effect of windowing settings on the images. A method to measure LCD using a model observer has previously been explored by Bellesi et al.^31^ using CTQA_cp (an automatic image quality analysis of CT images of the Catphan 600/504 phantom). Although the CTQA_cp can be a valid tool in analyzing LCD data, CTQA_cp does not work well on images obtained from 100‐mA s scans due to its intrinsic limitations and can only be used on Catphan phantom images.

Another objective method in LCD assessment is the statistical method. However, the method for calculating statistical LCD is limited to a few vendors, making it difficult for the practice to be widely accessible. The current study developed software to overcome these limitations. Using a GUI design that is user friendly, users are able to perform statistical LCD measurement quickly and conveniently. The images that can be measured are not limited to scans from certain scan parameters, phantoms, and scanners, because the software can be implemented on all DICOM format images as long as the image has a large enough uniform region to create a cell grid ROI of various sizes.

Figure 8 shows that the noise level affects the detectability of an object from the background. It was found that if noise level increases, then the target size also increases, as reported previously.^30^, ^31^ This is because the high levels of noise affect the shape of the sharp edge of the object, reducing the MDC especially for small objects.

We found that the MDC value was higher in the low‐contrast module than in the distance module because of the greater noise present in the low‐contrast module. This is because the low‐contrast module is composed of materials with a CT number of about 85 HU, whereas the distance module is composed of water with a CT number of about 0 HU. Thus, in the low‐contrast module, X‐ray absorption is higher compared to the distance module. As a result, the noise in the low‐contrast module is greater than in the distance module.

Our software was able to find the *C*–*D* curve for various homogeneous phantoms. Figure 10 shows that our algorithm can find the *C*–*D* curves and measure the MDCs from various phantoms. The resulting *C*–*D* curves have different characteristics because the phantoms were scanned with different input parameters (Table 2); however, they still follow the power‐law fit. The target size depends on the noise level within the image as mentioned earlier.

Our algorithm is also able to find the *C*–*D* curves and measure the MDCs from various scanners (Figure 11). As predicted, the resulting *C*–*D* curves have different characteristics because every scanner has specific characteristics and the phantom was scanned with different input parameters (Table 3). Clearly, the scan parameters used in the Siemens Emotion 16 produce lower noise, so the corresponding target size in the Siemens Emotion 16 scanner was smaller. Thus, the algorithm has advantages in terms of universality and flexibility.

In the current study, visual evaluation was conducted to evaluate the statistical LCD on the low‐contrast module of the ACR CT phantom. Table 4 indicates that visual evaluation shows that the object (with 0.6% MDC) that can be identified by the human observer is slightly larger than with statistical LCD. This is because human observation is very subjective and influenced by various factors, such as observers’ experience, and the window width and window level used. In this study, both the window width and window level were 100 HU. The use of a smaller window width might increase the visibility of objects by observers. However, the trends based on the two methods were the same. Expert radiologists will be included in the next study.

Our software can vary the number of grids freely, not just 9 × 9 pixels. (The effect of cell grid size on LCD results will be investigated in a future study.) One drawback of our system is that the center of the grid can only be moved up, down, right, and left, so that for some phantom images, homogenous areas cannot be detected accurately. The system needs to be improved, so that the determination of the center of the grid can be more flexible, depending on the phantom used.

In the implementation of statistical LCD, the user should have a comprehensive understanding of the process of image generation and image characteristics to avoid overestimating the LCD of the system (e.g., when a post processing filter is applied to suppress noise in an image). Although many filters are adaptive (such as the selective mean filter,^34^ bilateral filter,^35^ and non‐local mean filter^36^) and have capabilities to avoid smoothing real structures, adaptive filters use certain criteria to differentiate between noise and a real structure. If the contrast of the LCD object is below the threshold, the object will be treated as noise and smoothed by the filters. As the statistical method uses only uniform phantoms, the LCD always increases when the filters are applied. In contrary, if the measurement is carried out with a real low‐contrast phantom and a human observer, the implementation of the filters leads to a reduction in the LCD. Therefore, the implementation of statistical LCD makes it possible to get overestimated results. This illustrates the complexity of statistical LCD measurements. It may be overcome by using images containing LCD objects, subsequently developing a statistical LCD method with a priori knowledge of the size and location of the object. This will be conducted in an upcoming study.

## 5 CONCLUSION

We developed a GUI‐based program to automatically generate *C*–*D* curves based on statistical LCD of CT phantom images using multiple cell sizes. Our software calculated MDC characteristics for target sizes of 1.3–9.97 mm automatically. As expected, the MDC with respect to target size followed a power‐law relationship. It was found that higher noise levels resulted in a higher MDC for a target of the same size. The low‐contrast module image had a slightly higher MDC than the distance module image at the same slice thickness and ROI size. The minimum size of an object that can be detected by visual observation is slightly larger than the size using the automated method based on statistical LCD. Our algorithm can be used to test the LCD of most available phantoms and scanners with high flexibility.

## CONFLICT OF INTEREST

C.A. and A.N. are the developers of IndoQCT, a software for CT image quality evaluation. This software is not commercially available yet. Contact anam@fisika.fsm.undip.ac.id for more information.

## AUTHOR CONTRIBUTION

Choirul Anam and Ariij Naufal conceived the idea and developed the software. Toshioh Fujibuchi and Kosuke Matsubara provided images of phantoms. Choirul Anam and Geoff Dougherty wrote the paper. All authors approved the final version of the paper.

## Data Availability

Data are available on request from the authors.
